# Antiplatelet and Anti-Coagulation Therapy for Left-Sided Catheter Ablations: What Is beyond Atrial Fibrillation?

**DOI:** 10.3390/jcm12196183

**Published:** 2023-09-25

**Authors:** Martina Nesti, Fabiana Lucà, David Duncker, Francesco De Sensi, Katarzyna Malaczynska-Rajpold, Jonathan M. Behar, Victor Waldmann, Ahmed Ammar, Gianluca Mirizzi, Rodrigue Garcia, Ahran Arnold, Evgeny N. Mikhaylov, Jedrzej Kosiuk, Luigi Sciarra

**Affiliations:** 1 Fondazione Toscana G. Monasterio, 56124 Pisa, Italy; martina.nesti@tiscali.it (M.N.);; 2 Cardiology Department, Grande Ospedale Metropolitano, 89129 Reggio Calabria, Italy; 3 Hannover Heart Rhythm Center, Department of Cardiology and Angiology, Hannover Medical School, 30625 Hannover, Germany; 4 Cardiology Department, Misericordia Hospital, 58100 Grosseto, Italy; 5 Royal Brompton and Harefield NHS Foundation Trust, London SW3 6NP, UK; katarzyna.rajpold@gmail.com; 6 Georges Pompidou European Hospital, 75015 Paris, France; 7 Barts NHS Trust, London E13 8SL, UK; 8 Department of Cardiology, Ain Shams University, Cairo 11517, Egypt; 9 CHU de Poitiers, 2 Rue de la Milétrie, 86021 Poitiers, France; rodrigue_garcia@hotmail.fr; 10 Department of Cardiology, University of Poitiers, 15 Rue de l’Hotel Dieu, 86000 Poitiers, France; 11 National Heart and Lung Institute, Imperial College London, London SW7 2BX, UK; 12 Almazov National Medical Research Centre, 197341 Saint-Petersburg, Russia; e.mikhaylov@almazovcentre.ru; 13 Rhythmology Department, Helios Clinic Köthen, 06366 Köthen, Germany; 14 Department of Clinical Medicine, Public Health, Life and Environment Sciences, L’Aquila University, 67100 L’Aquila, Italy

**Keywords:** left-sided ablations, anticoagulation therapy, ventricular tachycardia, atrial tachycardia

## Abstract

**Aim:** International guidelines on the use of anti-thrombotic therapies in left-sided ablations other than atrial fibrillation (AF) are lacking. The data regarding antiplatelet or anticoagulation strategies after catheter ablation (CA) procedures mainly derive from AF, whereas for the other arrhythmic substrates, the anti-thrombotic approach remains unclear. This survey aims to explore the current practices regarding antithrombotic management before, during, and after left-sided endocardial ablation, not including atrial fibrillation (AF), in patients without other indications for anti-thrombotic therapy. **Material and Methods:** Electrophysiologists were asked to answer a questionnaire containing questions on antiplatelet (APT) and anticoagulation therapy for the following left-sided procedures: accessory pathway (AP), atrial (AT), and ventricular tachycardia (VT) with and without structural heart disease (SHD). **Results:** We obtained 41 answers from 41 centers in 15 countries. For AP, before ablation, only four respondents (9.7%) used antiplatelets and two (4.9%) used anticoagulants. At discharge, APT therapy was prescribed by 22 respondents (53.7%), and oral anticoagulant therapy (OAC) only by one (2.4%). In patients with atrial tachycardia (AT), before ablation, APT prophylaxis was prescribed by only four respondents (9.7%) and OAC by eleven (26.8%). At discharge, APT was recommended by 12 respondents (29.3%) and OAC by 24 (58.5%). For VT without SHD, before CA, only six respondents (14.6%) suggested APT and three (7.3%) suggested OAC prophylaxis. At discharge, APT was recommended by fifteen respondents (36.6%) and OAC by five (12.2%). Regarding VT in SHD, before the procedure, eight respondents (19.5%) prescribed APT and five (12.2%) prescribed OAC prophylaxis. At discharge, the administration of anti-thrombotic therapy depended on the LV ejection fraction for eleven respondents (26.8%), on the procedure time for ten (24.4%), and on the radiofrequency time for four (9.8%), with a cut-off value from 1 to 30 min. **Conclusions:** Our survey indicates that the management of anti-thrombotic therapy surrounding left-sided endocardial ablation of patients without other indications for anti-thrombotic therapy is highly variable. Further studies are necessary to evaluate the safest approach to these procedures.

## 1. Introduction

Catheter ablation (CA) procedures are associated with a potential threat of thromboembolic complications in patients without other indications for anti-thrombotic therapy. Catheter instrumentation activates the clotting cascade and, consequently, increases the risk of thrombus formation [1,2], particularly in the case of procedures performed on the left side of the heart. Most of the available data are focused on periprocedural anticoagulation regimens and describe CA procedures for atrial fibrillation (AF) [3,4,5]. However, the anticoagulation protocols during other left-sided ablation procedures are not well assessed, particularly in complex ablations with extensive radiofrequency applications. The purpose of our survey was to address the contemporary clinical practices of electrophysiologists in antiplatelet (APT) and anticoagulation therapy (OAC) for left-sided endocardial CA, other than AF, in patients without other indications for antithrombotic therapy.

## 2. Methods

An online questionnaire consisting of multiple-choice questions was prepared and sent via SurveyMonkey to centers among electrophysiologists’ scientific network that performed left-sided ablation. The responses were collected from 1 February 2019 to 15 March 2019. This study complied with the European General Data Protection Regulation law. All centers that took the survey agreed to participate in the study.

### 2.1. Data Collected

The questionnaire collected information on antithrombotic management before, during, and after left-sided endocardial CA procedures, except for AF, in patients without other indications for anti-thrombotic therapy. Left-sided ablation for atypical atrial flutter was also excluded. The left-sided endocardial CA procedures that were evaluated included CA of an accessory pathway (AP), atrial tachycardia (AT), ventricular tachycardia (VT) without known structural heart disease (SHD), and VT in SHD. Antithrombotic therapy included all pharmacological agents used to treat or avoid thromboembolism, including vitamin K antagonists (VKA), novel oral anticoagulants (NOAC) as well as APTs such as aspirin and P2Y12 inhibitors.

The questionnaire also collected information about the type of center (academic vs. public vs. private), the country of location, the number of procedures/year, the number of left-sided ablation procedures/year, and the number of electrophysiologists working in the EP lab.

The complete questionnaire is provided in Appendix A.

### 2.2. Statistical Analysis

Categorical data were reported as numbers and percentages. The mean (standard deviation [SD]) and the median (interquartile range [IQR]) have been used for the description of normally and non-normally distributed data, respectively. All data were analyzed using SPSS v 20.0 (SPSS Inc., Chicago, IL, USA). The authors had full access to the data and take full responsibility for its integrity. All authors have read and agreed to the manuscript as written.

## 3. Results

The centers were contacted to participate to the survey through EHRA database. Forty-one centers from 15 countries responded (63.4% university hospitals, 24.4% public hospitals, and 12.2% private hospitals) (Table 1).

The median number of EP procedures per center was in the range of 503 to 441 per year. The number of left-sided procedures per year was in the range of 37 to 21 (not including AF CA).

### 3.1. Accessory Pathway

**Figure 1:**
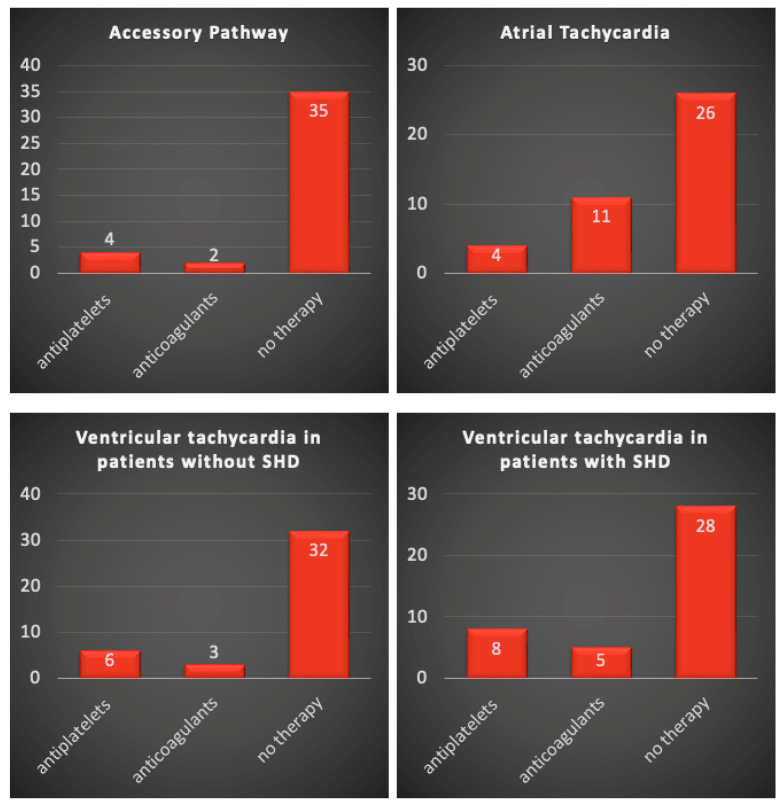
Anti-thrombotic management before ablation. SHD: structural heart disease.

**Figure 2:**
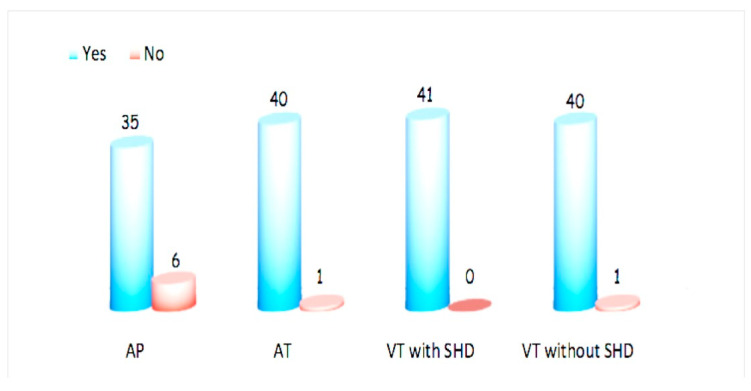
Use of heparin during ablation. AP: accessory pathway, AT: focal atrial tachycardia, VT: ventricular tachycardia, SHD: structural heart disease.

**Figure 3:**
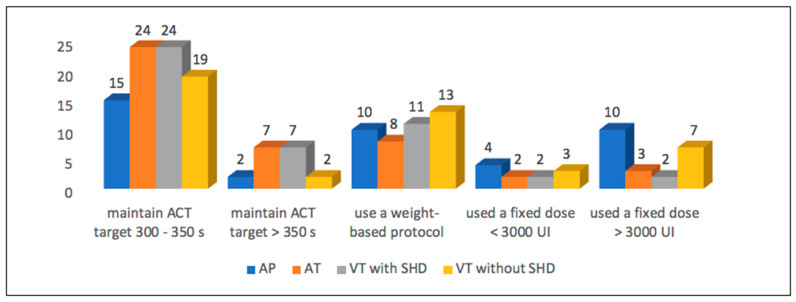
Heparin dosage during ablation. AP: accessory pathway, AT: atrial tachycardia, VT: ventricular tachycardia, SHD: structural heart disease, ACT: activated clotting time.

**Figure 4:**
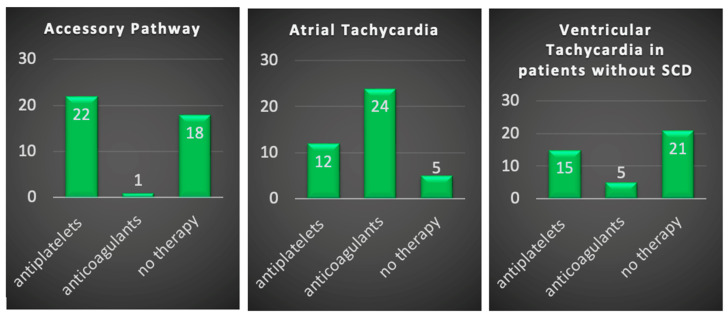
Anti-thrombotic management at discharge. SHD: structural heart disease.

Before CA, the majority of respondents (35, or 85%) did not use antithrombotic therapy. Only four respondents (9.7%) administered APT and two (4.9%) administered OAC (Figure 1). During CA, heparin was used by 85.4% (Figure 2) to maintain the ACT target of 300–350 s in 36.6% of cases (Figure 3). Heparin was used to irrigate the sheaths by 22 respondents (53.7%). After CA, APT was prescribed by twenty-two respondents (53.7%) and OAC only by one (2.4%) (Figure 4).

### 3.2. Atrial Tachycardia

Before CA, APT prophylaxis was recommended by only four respondents (9.7%) and OAC by eleven (26.8%) (Figure 1). During the procedure, almost all respondents (40, or 97.6%) used heparin (Figure 2), and an ACT target of 300–350 s was adopted in 58.5% of cases (Figure 3). The sheaths were routinely irrigated with continuous intravenous heparin by 25 respondents (61%). After CA, APT was recommended by 12 respondents (29.3%) and OAC by 24 (58.5%) (Figure 4).

### 3.3. Ventricular Tachycardia in Patients without Structural Heart Disease

Before CA, only six respondents (14.6%) suggested antiplatelets and three (7.3%) suggested anticoagulation prophylaxis (Figure 1). During ablation, almost all respondents (40, or 97.6%) used heparin (Figure 2), maintaining an ACT target of 300–350 s in 46.3% of cases (Figure 3). Continuous intravenous heparin was used by 22 respondents (53.7%) to irrigate the sheaths. After CA, APT was recommended by fifteen (36.6%) and OAC by five respondents (12.2%) (Figure 4).

### 3.4. Ventricular Tachycardia in Patients with Structural Heart Disease

APT and OAC prophylaxis before CA were prescribed by eight (19.5%) and five (12.2%) respondents, respectively (Figure 1). Conversely, the intraprocedural use of heparin was adopted by all respondents (Figure 2), maintaining an ACT target of 300–350 s in 58% of cases (Figure 3). The sheaths were routinely irrigated with continuous intravenous heparin by 26 respondents (63.4%). After CA, the choice of administering APT was based on the left ventricular ejection fraction (LVEF), the procedure time, and the radiofrequency time, with a cut-off value ranging from 1 to 30 min for eleven (26.8%), ten (24.4%), and four (9.8%) respondents, respectively. (Figure 1). During CA, all respondents used heparin (Figure 2), maintaining an ACT target of 300–350 s in 58% of cases (Figure 3). The sheaths were routinely irrigated with continuous intravenous heparin by 26 respondents (63.4%). After CA, the administration of APT depended on the left ventricular ejection fraction (LVEF) for eleven respondents (26.8%), on the procedure time for ten (24.4%), and on the radiofrequency time for four (9.8%), with a cut-off value ranging from 1 to 30 min.

## 4. Discussion

Nowadays, CA is considered the strategy of choice for a wide range of arrhythmias in light of its high percentage of success and its low rate of complications [6]. With regard to thromboembolic complications, it is worth mentioning that manipulating catheters and simultaneously performing lesions during procedures may increase thrombotic risk, particularly in left heart procedures [7,8,9,10].

An incidence of cerebral embolism (CE) and peripheral arterial embolism of 0.46–0.06% in left-sided CS has been reported by Hindricks [8].

In the MERFS registry [8], which analyzed 1715 subjects who underwent right-sided ablation (AV node re-entrant tachycardia or AV junction ablation), the rates of CE, pulmonary embolism (PE), and venous thrombosis (VTE) were 0.06%, 0.23%, and 1.04%, respectively. On the other hand, the percentages of pericardial tamponade (PT), pericardial effusion (PEff), and major bleeding (MB)/hematoma have been reported as 0.17%, 0.41%, and 0.11%, respectively.

In the NASPE registry [11], among 2142 adults who underwent right-sided ablation, the rates of thrombo-embolism, PT, and MB/hematoma have been shown to be 0.14%, 0.09%, and 0.28%, respectively. In contrast, no embolic events have been reported after the CA of left free-wall accessory pathways in 418 adults.

In Atakr Multicenter’s registry [12], it has been observed that thromboembolic events occurred in 0.7% and 1.1% of patients who underwent right-sided and left-sided CA, respectively, in the absence of other risk factors for systemic embolization.

In comparison, rates of PT, PEff, and MB/hematoma of 0.6%, 1.9%, and 3.5%, respectively, were described [12].

Furthermore, embolic complications after CA procedures in patients with VT seem to be lower in the absence of SHD compared to patients with structural abnormalities.

However, an anticoagulation strategy is usually used during CA procedures, consisting of administering a venous bolus of heparin (50–100 U/kg) followed by a heparin infusion with the aim of maintaining an ACT above 300 s [13,14,15].

Nevertheless, the intraprocedural risk of a systemic thromboembolic event is significantly lowered by the intravenous administration of heparin or bivalirudin [16]. Despite this, the post-procedural risk remains considerable, and it should be accurately evaluated [16]. Indeed, it has been shown that cerebral events, including subclinical ones, cause long-term neurocognitive impairment [17].

However, data regarding either the APT or the OAC approach after CA are limited.

Our survey sheds light on the fact that considerable variation exists in the management of OAC and APT surrounding left-sided non-AF endocardial CA in patients without other indications for anti-thrombotic therapy.

However, it should be highlighted that for some indications, the respondents largely agreed not to use antithrombotic medication, whereas the indications varied considerably for other procedures. During left-sided electrophysiological procedures, due to an increase in the thrombophilic state, a three-fold increase in the incidence of thromboembolic complications (1.8–2%) was observed, compared with an overall rate of only 0.6% when all CA procedures are considered [18]. Despite this important data that differs from AF indications [4], no guidelines indicate the correct choices in this setting. The only indication was given by the consensus document published in 2015 [18] and by a recent consensus on ventricular arrhythmias [19].

A previous survey about the prevention of VTE after EP procedures was conducted; however, it described only right-sided ablation [20]. To our knowledge, this is the first survey about left-sided ablations.

### 4.1. Accessory Pathway

Patients undergoing accessory pathway (AP) CA are more likely to be young, without risk factors for thromboembolic events, or those who are at low risk. Only a single catheter in the left atrium (LA) or left ventricle (LV) is commonly used; moreover, the ablation (CA) is usually focal, resulting in much shorter total CA times and less time spent in the left atrium. For this reason, Sticherling et al. [18] recommend neither anti-thrombotic prophylaxis before AP ablation, nor the post-interventional use of OAC or APT.

Moreover, previous studies reported an incidence of 0.46–2% [8,16] of thromboembolic events related to AP ablation and, recently, Głowniak et al. documented the presence of silent cerebral infarcts after AP ablation [21]. Thakur et al. [22] reported that 2% of embolic events were late incidences in left-sided accessory pathways CA, in spite of the intraprocedural administration of heparin followed by APT for 3 months after CA.

For this reason, continuous flushing of the sheaths and antithrombotic therapy (with 5000–15,000 U or 90–200 U/kg of intravenous sodium heparin followed by 1000 U/h) are advised during the procedure to avoid thrombus formation [18].

Our survey showed a different scenario: 15% of surveyed participants used antithrombotic prophylaxis before CA of the accessory pathway and 50% used it after CA. On the other hand, the heparin dose was mainly driven by the activated clotting time (ACT) value, and only half of the participants irrigated the sheaths during procedures.

### 4.2. Atrial Tachycardia

In contrast to patients with atrial flutter, who are thought to have the same risk of thromboembolism as patients with AF [23], there is no clear data regarding thromboembolic risk in patients with FAT. In our survey, only about a quarter of respondents used OAC in patients with FAT prior to the procedure, and about one-fifth recommended APT. During CA procedures, almost all the participants were heparinized. The respondents administered heparin to their patients based on ACT control, and over half of them also used heparin in side flushes. Heparinization with ACT > 300 s is a standard of care in left-sided CA procedures according to the most recent European guidelines for AF management [24]; however, it was not specified whether the continuous irrigation of the sheaths further reduced the risk of thromboembolic complications.

After the CA procedure, slightly more than half of the participants recommended OAC, and a quarter preferred to give antiplatelet agents. The rationale for using OAC after AF ablation included the risk of arrhythmia in the blanking period and the phenomenon of atrial stunning following sinus rhythm restoration [25]. Again, there is no data on the prevalence of LA appendage thrombus after left-sided FAT CA. More data is needed to understand if the risk of thromboembolic events in FAT is similar to that in AF, and hence if respective antithrombotic therapy should be warranted.

### 4.3. Ventricular Tachycardia in Patients without Structural Heart Disease

VT can also occur in a structurally normal heart [26]. Idiopathic VTs (the most common type) are typically monomorphic because they originate from a single focus, and can be ablated by the limited delivery of radiofrequency energy to the site of origin of the arrhythmia [15]. Probably due to the limited area of CA, the risk of thromboembolism in patients without SHD undergoing VT ablation is lower than in patients with SHD [27], but data about the correct management of antithrombotic therapy in these procedures are not available, and there is a large variability among participants. 

In a survey regarding intraprocedural anticoagulation among the writing committee members of the EHRA/HRS Expert Consensus on Catheter Ablation of Ventricular Arrhythmias [28], for idiopathic VA, 48% of the respondents used ACT levels longer than 250 s, 39% longer than 300 s, and 13% longer than 350 s. However, a clear distinction was not made between VT with and without SHD regarding antithrombotic therapy after CA. Our data confirmed the use of heparin during ablation. However, a difference in terms of ACT targets has been reported. Indeed, the data from our survey indicated that the achieved ACT values were higher, ranging from 300 to 350 s (Figure 2).

### 4.4. Ventricular Tachycardia in Patients with Structural Heart Disease (SHD)

Our results showed that anti-thrombotic therapy was variable in patients undergoing LV substrate ablation.

According to the 2019 HRS/EHRA/APHRS/LAHRS Consensus [19], antithrombotic therapy should not be adopted before CA; however, this point is not generally agreed upon.

Regarding anticoagulation during the CA procedure, previous authors have suggested different protocols: a bolus of at least 5000 U after the insertion of sheaths followed by a 1000 U/h heparin infusion without intra-procedural ACT monitoring [29], or strict ACT monitoring with the target values of 200–250 s [30]. In contrast, according to the consensus document, after sheath insertion, the administration of a bolus of intravenous heparin (bolus dose empirically 5000–10,000 U or 50–100 U/kg) should be followed by a continuous infusion of 1000–1500 U/h in order to maintain an ACT level of 300 s. Our data are not consistent with these indications. Indeed, the ACT target is higher (300–350).

Regarding post-procedural anticoagulation management, in our survey, APT was prescribed after CA by 53.6% of respondents and OAC by 31.7%.

However, there are no conclusive data in this sense [31,32], and the choice of APT after CA depends on the physician [7] (Table 2).

Another important part of the data to consider is the role of OAC with warfarin or NOACs for patients who received extensive areas of CA, or those who are at increased risk of thromboembolism. In a paper by Reddy et al. [33], and in the Multicenter Thermocool Ventricular Tachycardia Ablation Trial [34], the choice between VKA or aspirin after a VT ablation depended on the extension of the CA area (Table 2). In the SMASH VT study [33], OAC was continued for 4 to 6 weeks (providing that they had more than five CA lesions). In the Multicenter Thermocool Ventricular Tachycardia Ablation Trial [34] patients received OAC for 3 months in cases in which CA was performed on over 3 cm of the lesion area.

The EHRA/HRS Expert Consensus on Catheter Ablation of Ventricular Arrhythmias [35], published in 2009, recommended 6–12 weeks of warfarin after CA over large endocardial areas. However, the latest version [19] suggested that anticoagulation is reasonable after less extensive endocardial VT ablation, or with OAC after extensive endocardial VT ablation (classes of recommendation IIa and IIb, and level of evidence C, respectively) even without a specific indication regarding the timing.

More recently, according to Shivkumar et al., it should be advisable to continue OAC after VT CA for at least 4 weeks in these patients and this indication should be extended to all cases of extensive ablation. With regard to long-term OAC, the choice should be based on whether preexisting indications exist or not [15]. Despite this evidence, in our survey, only four respondents followed this indication. More respondents decided to prescribe OAC or APT therapy according to the LV ejection fraction (26.8%).

## 5. Limitations

This study has several limitations. These data represent the most current practices among some electrophysiologists, which may not represent the standards of practitioners in other countries, or those in other settings. Moreover, voluntary participation can represent a selection bias. It should be noted that this may be exacerbated by the fact that the centers and not the individual doctors received the survey. The use of anti-thrombotic therapy depends on the radiofrequency time and the LV ejection fraction, but no data are available on the drugs used. Moreover, the choice of treatment in clinical practice is likely to be influenced by diverse clinical factors.

## 6. Conclusions

Our survey showed that there is considerable variation in the management of anti-thrombotic therapy surrounding left-sided non-AF endocardial CA in patients without other indications for anti-thrombotic therapy. Further studies are necessary to evaluate the optimal approach to these procedures.

## Figures and Tables

**Table 1:** Countries of responders.

Italy	12 (29.3%)
Russia	7 (17.1%)
France	3 (7.3%)
Turkey	3 (7.3%)
Austria	2 (4.9%)
Belgium	2 (4.9%)
Croatia	2 (4.9%)
Greece	2 (4.9%)
Romania	2 (4.9%)
United Kingdom	2 (4.9%)
Germany	1 (2.4%)
Poland	1 (2.4%)
Spain	1 (2.4%)
Saudi Arabia	1 (2.4%)

**Table 2:** Antithrombotic management after ablation of ventricular tachycardia in structural heart disease.

Study	Antithrombotic Strategies Post Ablation	Duration of Antithrombotic Therapy
Chen et al. (1996) [28]	None	
Calkins et al. (2000) [31]	No indication	
Reddy et al. (2007) [33]	- VKA	4–6 weeks
	- Aspirin if <5 ablation lesions	
Stevenson et al. (2008) [34]	- VKA for ablation area >3 cm	3 months
	- aspirin 325 mg for ablation area <3 cm	
Aliot et al. (2009) [35]	- VKA for large ablation area	6–12 weeks
Kuck et al. (2010) [32]	No indication	
Cronin et al. (2019) [19]	- VKA for extensive ablation	No indication
	- antiplatelet agent for less extensive ablation	
Shivkumar et al. (2019) [15]	- OAC for at least 4 weeks	Based on preexisting indications

VKA: vitamin K antagonist; OAC: oral anticoagulant therapy.

## Data Availability

Not applicable.

## Appendix A

Questionnaire:

1. Center characteristic
  a. Type of institution (University Hospital, Public Hospital, Private Hospital)
  b. Country
  c. Number of procedures/year
  d. Number of left-sided ablation procedures/year (accessory pathway, atrial tachycardia, ventricular tachycardia)

2. Before left-sided ablation—In the absence of other indications for anti-thrombotic therapy...
  a. Do you use antiplatelet prophylaxis in:
    - the accessory pathway? yes/no
    - atrial tachycardia? yes/no
    - ventricular tachycardia with structural heart disease? yes/no
    - ventricular tachycardia without structural heart disease? yes/no
  b. Do you use anticoagulation prophylaxis in:
    - the accessory pathway? yes/no
    - atrial tachycardia? yes/no
    - ventricular tachycardia with structural heart disease? yes/no
    - ventricular tachycardia without structural heart disease? yes/no

3. During left-sided ablation—In the absence of other indications for anti-thrombotic therapy
  a. Do you use heparin in:
    - the accessory pathway? yes/no
    - atrial tachycardia? yes/no
    - ventricular tachycardia with structural heart disease? yes/no
    - ventricular tachycardia without structural heart disease? yes/no
  b. Do you use heparin to irrigate the sheath introducers? yes/no
  c. For the heparin dose do you
    - maintain ACT target 300–350 s
    - maintain ACT target >350 s
    - use a weight-based protocol
    - use a fixed dose < 3000 Units
    - use a fixed dose > 3000 Units

4. After ablation—In the absence of other indications for anti-thrombotic therapy...
  a. Do you prescribe antiplatelet therapy after the ablation of
    - the accessory pathway? yes/no
    - atrial tachycardia? yes/no
    - ventricular tachycardia with structural heart disease? yes/no
    - ventricular tachycardia without structural heart disease? yes/no
  b. After the ablation of a ventricular tachycardia with structural heart disease, does the use of anti-thrombotic therapy depend on the procedure time? yes/no
  c. After ablation of ventricular tachycardia with structural heart disease, does the use of anti-thrombotic therapy depend on the radio frequency time? yes/no
  d. If yes, which is the cut-off (minute of radiofrequency)?
  e. After ablation of ventricular tachycardia with structural heart disease, does the use of anti-thrombotic therapy depend on the LV ejection fraction? yes/no

5. Is the anti-thrombotic protocol the same among physicians at the same center? yes/no
